# Novel roles of PRK1 and PRK2 in cilia and cancer biology

**DOI:** 10.1038/s41598-020-60604-3

**Published:** 2020-03-03

**Authors:** Hitesh Patel, Jun Li, Ana Herrero, Jakob Kroboth, Adam Byron, Alex Von Kriegsheim, Valerie Brunton, Neil Carragher, Toby Hurd, Margaret Frame

**Affiliations:** 1 University of Edinburgh, Edinburgh Cancer Research UK Centre, Institute of Genetics and Molecular Medicine, Crewe Road South, Edinburgh, United Kingdom; 2 University of Sussex, Sussex Drug Discovery Centre, School of Life Sciences, Brighton, BN1 9QJ United Kingdom

**Keywords:** Breast cancer, Cell polarity

## Abstract

PRK1 and PRK2 are two closely related AGC-family serine/threonine protein kinases. Here we demonstrate novel roles for them at cilia and in cancer biology. In both instances serum withdrawal leads to increased activating PRK1 and PRK2 phosphorylation (pPRK1/pPRK2) and their depletion results in reduced spheroid growth. pPRK1/pPRK2 localise to the transition zone of cilia and their co-depletion results in reduced cilia size, impaired planer polarity and impaired cilia associated signalling. High PRK2 (but not PRK1) expression correlates with poor outcome in patients with basal-like/Triple Negative (TN) Breast Cancer (BC) where there is also higher expression relative to other BC tumour subtypes. In agreement, depletion of PRK1 and PRK2 in mouse TNBC cells, or CRISPR/Cas9 mediated deletion of PRK2 alone, significantly reduces cell proliferation and spheroid growth. Finally proteomic analysis to identify PRK2 binding partners in mouse TNBC cells revealed proteins that are important for both cilia and BC biology. Taken together these data demonstrate novel roles for PRK1 and PRK2 at cilia and in BC biology and in the case of PRK2 in particular, identifies it as a novel TNBC therapeutic target.

## Introduction

Protein kinase C related kinases (PRK1-3/PKN1-3) are a group of 3 related serine/threonine protein kinases that reside within the AGC family of kinases (Supplemental Fig. 1A). PRK1 and PRK2 are more closely related to each other in terms of sequence identity and domain structure, while PRK3 is more distant (Supplemental Fig. 1B). All three isoforms in mouse contain a serine/threonine kinase domain and 3 Rho binding (HR1) domains, while PRK1 and PRK2 also contain a Ca^2+^ dependent membrane targeting (C2) domain. Activities of PRK1 and PRK2 are thought to be regulated by a combination of auto-phosphorylation and phosphorylation by PDPK1 and, by the binding of small RHO family of GTPases and lipids^1–8^. PRK1 and PRK2 have been implicated in a number of physiological processes^9–13^; however, they remain relatively understudied and uncharacterised in terms of molecular and spatial function, as well as in the context of disease. A number of substrates have been identified for PRK1 (e.g. MAPT, VIM, NEFH/L/M and HDAC5/7/9)^14–18^ and PRK2 (CTTN and HDAC5)^18,19^. These consist of a diverse collection of proteins that fulfil a variety of cellular functions.

**Figure 1:**
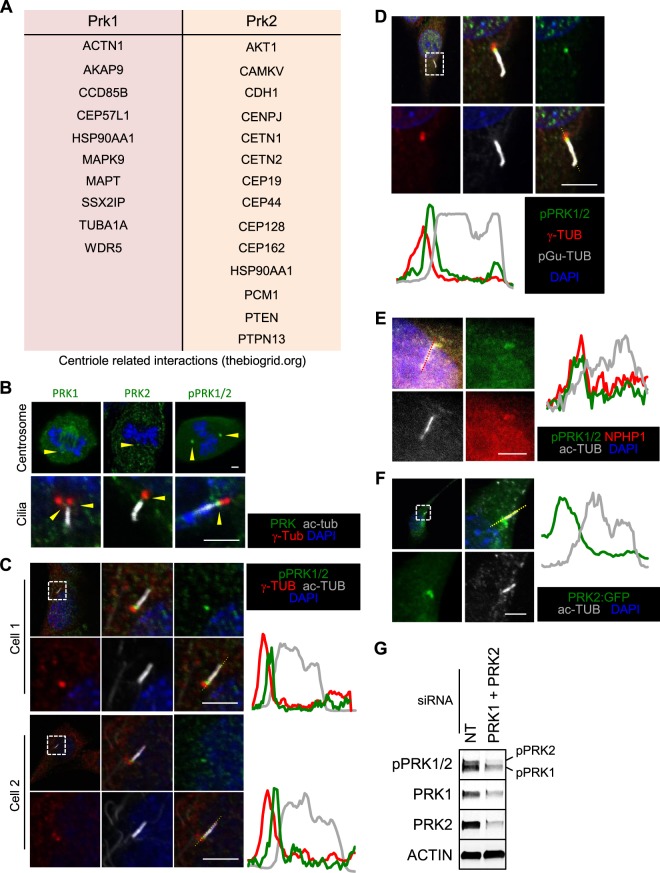
PRK1 and PRK2 interact with centriole associated components and structures and localise to the transition zone of cilia. (**A**) Centriole associated proteins stated (thebiogrid.org) to interact with PRK1 or PRK2. (**B**) Immunofluorescence images of centriolar structures (centrosomes and cilia). (**C–E**) Immunofluorescence images of pPRK1 and pPRK2 (pPRK1/2) at the base of cilia with various other cilia associated markers. Line traces along the cilium are shown to demonstrate localization to the transition zone. (**F**) Immunofluorescence image of a NIH3T3 cell exogenously expressing PRK2 fused to GFP (PRK2:GFP). Scale bar represents 2 µm. (**G**) Western blot analysis of lysate from cells treated with non-targeting (NT) or PRK1 and PRK2 (PRK1 + PRK2) siRNA. Unless stated otherwise, n ≥ 3.

To begin to understand how PRK1 and PRK2 contribute to disease, we performed a literature search for known binding partners. This revealed RHO, RAC and PDPK1^4,5,20^, well established as key regulators of the actin cytoskeleton (Rho and Rac) and proliferation (PDPK1), which are often mis-regulated in cancer. Search of protein interaction databases (e.g. thebiogrid.org), revealed a diverse array of other proteins that interact with PRK1/PRK2, suggesting that the roles PRKs play in cellular processes and diseases is diverse and, at least in part, through protein interactions (Supplemental Fig. 1C). Several of these putative interactions suggest a link to centriole function and/or localization to the centriole (Fig. 1A). Centrioles are microtubule derived structures that play a critical role in multiple cellular processes, including cell division, cell migration, polarity and ciliogenesis^21^, all of which are processes that contribute to ciliopathies and cancer. Indeed a number of studies have been conducted that explore the link between cilia and cancer biology in various tissues^22^, including breast^23,24^. Cilia are organelles that act as physical and chemical sensors that most normal breast tissue types have the ability to form, including fibroblasts (at 42% incidence), luminal epithelial (at 10% incidence) and myoepithelial (49%)^24^. These studies find that though the ability to form cilia is largely lost early during cancer development, a specific subtype of cancer cells, such as Basal-like cells in breast cancer, retain it at a low incidence. Basal-like cells are representative of the Triple Negative Breast Cancer (TNBC) subtype of tumours that generally have poor clinical outcome. In addition a number of important signalling pathways in cancer such as those that regulate cell growth (ERK/MAPK) and differentiation (Hedgehog) are regulated by cilia.

Here we describe novel roles for PRK1 and PRK2 at cilia and in cancer biology. In both ciliating and non-ciliating (cancer) cells serum withdrawal leads to increased activating PRK1 and PRK2 phosphorylation while PRK2 depletion/loss results in reduced spheroid growth. In ciliated cells, phosphorylated PRK1 and PRK2 localise to the transition zone of cilia and their co-depletion results in impaired cilia and associated phenotypes. We show that high PRK2 (but not PRK1) expression correlates with poor outcome in patients with Basal-like/TNBC and that generally higher PRK2 expression and sensitivity to its depletion is seen in Basal-like/TNBC cell lines. Finally, PRK2 associates with proteins that are important for both cilia and breast cancer cell biology. Taken together, these data highlight novel roles for PRK1 and PRK2 at cilia and in breast cancer cell biology.

## Results

### PRK1 and PRK2 associate with centriolar components and structures and in particular, localise to the transition zone of cilia

Protein kinase C related kinases (PRK1-3/PKN1-3) are three closely related kinases that reside within the AGC family of kinases (Supplemental Fig. 1A). In mouse PRK1 and PRK2 are most closely related to each other (61.04% sequence identity), with both containing a serine/threonine kinase domain, three Rho binding domains (HR1) and a Ca2+ dependent membrane targeting (C2) domain (Supplemental Fig. 1B). PRK3 is the more distantly related family member, sharing ~53% sequence identity with the others and lacks a discernible C2 domain. Apart from their already published binding partners, Rho, Rac and Pdpk1, a number of other proteins have been reported to bind PRK1 and PRK2 in protein interaction databases such as thebiogrid.org (Supplemental Fig. 1C). Of these potential binding partners, a significant portion (10 out of 64 (~15.6%) for PRK1 and 14 out of 90 (~15.6%)) are linked to centriole function (Fig. 1A), which is important in diseases such as cancer and ciliopathy.

PRK2 activity has recently been implicated in a rare and newly established ciliopathy named STAR syndrome^25,26^ and as such we first determined whether PRK2 and/or PRK1 localised to centriolar structures and in particular to cilia (Fig. 1B). The phosphorylated forms of PRK1 and PRK2 - pPRK1/pPRK2 - showed localisation to centrosomes and to the base of cilia (Fig. 1B,C). We co-stained anti-pPRK1/pPRK2 and compartment specific cilia markers such as anti-γ-tubulin (centrioles), anti-acetylated-tubulin (cilia axoneme) and anti-polyglutamated-tubulin (basal body) and found that pPRK1/pPRK2 did not precisely localise with any of those markers within cilia, but instead localised to the transition zone at cilia (Fig. 1C,D). This was further confirmed by the co-localisation of pPRK1/pPRK2 with NPHP1^27^ – a known transition zone localising protein (Fig. 1E). Though data from NIH3T3 cells is shown here, similar localization (i.e. at the base of cilia) was observed in mouse embryonic fibroblasts (MEFs) and mouse Inner Medullary Collecting Duct-3 cells (mIMCD3) cells (Supplemental Fig. 1D). To confirm the localisation of PRK2, PRK2 fused to GFP (PRK2:GFP) was exogenously expressed in cells (Fig. 1F). PRK2-GFP also localised to the base of cilia similar to anti-PRK1, anti-PRK2 and anti-pPRK1/pPRK2 antibody staining, confirming the localisation.

The anti-pPRK1/pPRK2 antibody recognises phosphorylated threonine 774 and threonine 861 on PRK1 and PRK2, respectively, which are thought to represent the active forms of the kinases. Authenticity of the pPRK1/pPRK2 antibody was confirmed by siRNA mediated co-depletion of PRK1 and PRK2, which resulted in a reduction in the pPRK1/pPRK2 signal (Fig. 1G). Due to the potential implication for diseases such as ciliopathies and potentially to cancer, we next characterised the possible roles of pPRK1/pPRK2 at cilia and addressed whether they contribute to signalling and behavioural properties of cancer cells.

### PRK1/PRK2 are activated by the absence of serum and contribute to cilia size and signalling

**Figure 2:**
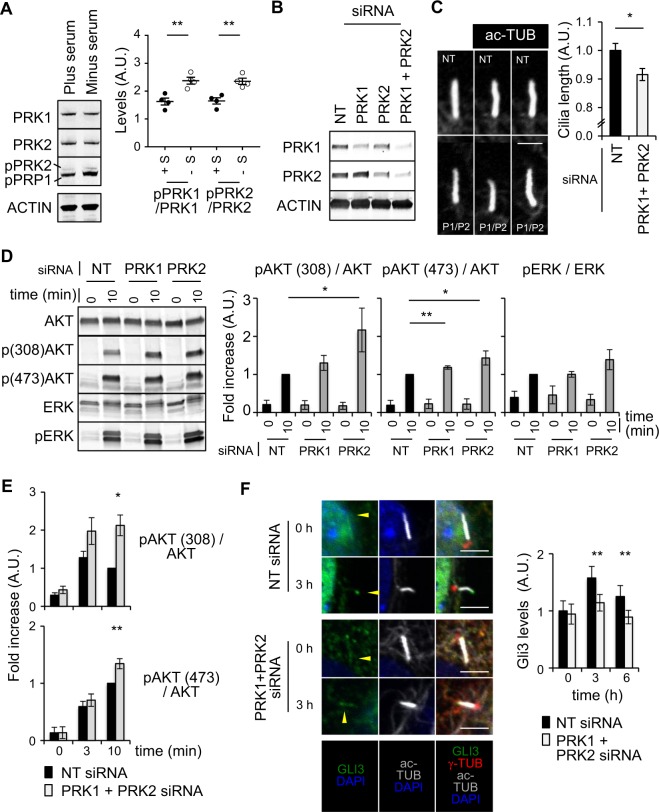
PRK1/PRK2 are activated by the absence of serum and contribute to cilia size and signaling. (**A**) Western blot analysis (left) and quantification (right) of lysate from cells in the presence (plus, +) or absence (minus, −) of serum. (**B**) Western blot analysis of lysate from cells treated with siRNA showing knockdown of PRK1, PRK2 and PRK1 + PRK2. (**C**) Representative images of cilia (left, top panel are NT and bottom panel are PRK1 + PRK2 siRNA treated cells, respectively) and quantification of their length (right) (>30 cilia were measured for each condition and scale bar represents 1 µm.). (**D**) Western blot analysis (left) and quantification (right) of lysate from serum starved cells treated with siRNA and stimulated with PDGF-AA. (**E**) Quantification of Western blot analysis of lysate from siRNA mediated PRK1 + PRK2 co-depleted cells stimulated with PDGF-AA. (**F**) Immunofluorescence images (left) of cilia from serum starved cells treated PRK1 + PRK2 siRNA and stimulated with purmorphamine (scale bar represents 2 µm). Quantification of the Gli3 intensity at the tip of cilia (>30 cilia for each condition measured) is shown on the right. NIH3T3 cells were used. Unless stated otherwise, n ≥ 3 ± s.e.m. shown.

In NIH3T3 cells (and other cells that have cilia), cilia formation is induced upon serum withdrawal. We found that while PRK1 phosphorylation was enhanced by serum-starvation, PRK2 levels were unaffected (Fig. 2A). This data suggests that PRK1/2 is not activated downstream of conventional growth factor signalling pathways, but instead may be activated upon initiation of ciliogenesis following serum withdrawal. Furthermore, PRK1/2 localization to the transition zone of cilia implies they have functions that originate there.

We examined if PRK1 and PRK2 were required for cilia formation by depleting both in NIH3T3 cells using siRNA and determining whether cilia formation or cilia length were affected (Fig. 2B). We found that cilia formation per se was unaffected, but that cilia size was reduced by approximately 10% in the PRK1 and PRK2 co-depleted cells when compared to cilia of non-targeting (NT) siRNA-treated control cells (Fig. 2C); the significance of this reduction in cilia length is unknown. We next measured two signalling outputs that are dependent on cilia function, PDGF-AA and Hedgehog signalling (Fig. 2D–F)^28,29^. In serum-starved control cells, PDGF-AA stimulation leads to increased pAKT (at residues 308 and 473) and pERK1/2 (p(Thr202/Tyr204)-ERK1 and p(Thr185/Tyr187)-ERK2) by 10 minutes (Fig. 2D). In PRK1-depleted cells only pAKT (473) showed a small but consistent enhancement, while PRK2 depletion resulted in enhanced pAKT (at both 308 and 473 residues). Co-depletion of PRK1 and PRK2 resulted in an increase of the phosphorylated forms of both Ser473- and Thr308-AKT (Fig. 2E). Stimulated pERK1/2 levels showed no consistent difference upon depletion of either PRK1 and/or PRK2 compared to control (Fig. 2D).

We also addressed whether PRK1 and/or PRK2 interacted with AKT in serum starved and stimulated cells (Supplemental Fig. 2A) by conducting co-immuno-precipitations. In starved cells AKT is in complex with PRK2, while upon stimulation for 10 min, there is a reduction in the AKT-PRK2 complex. There was no change in the AKT-PDPK1 complex.

Hedgehog stimulation of serum starved cells induces GLI2 and GLI3 protein accumulation at the tips of cilia^30^. Here the extent of GLI3 accumulation at cilia tips was measured using immunofluorescence (Fig. 2F). Serum starved PRK1- and PRK2-co-depleted cells displayed a decrease in GLI3 at the tips of cilia after 3 and 6 h of stimulation compared to control cells. Together, these data suggest that PRK1/2 functions to moderate cilia-mediated responses to extracellular ligands (such as PDGF-AA and SHH) as their co-depletion results in enhanced pathway activation.

### PRK1 and PRK2 co-depletion impairs planer polarity and anchorage independent growth

**Figure 3:**
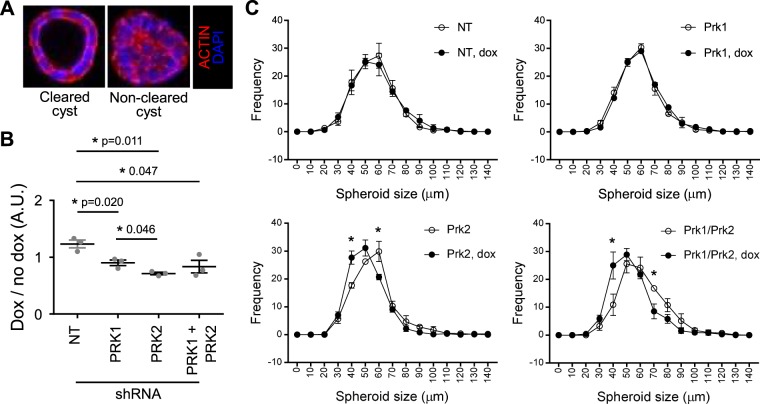
PRK1 and PRK2 co-depletion impairs planer polarity and anchorage independent growth. (**A**) Example of cleared and non-cleared mIMCD3 spheroid structures. (**B**) Quantification of cleared spheroids formed by mIMCD3 cells expressing doxycycline (dox) inducible shRNA constructs ± dox (>100 cysts quantified for each condition). (**C**) Distribution of the different spheroid diameters formed in B. Unless stated otherwise, n ≥ 3 ± s.e.m. shown.

One specific trait shared by many of the proteins that localise to the transition zone of cilia is their involvement in the establishment of both planar and apico-basal polarity^31,32^. mIMCD3 cells are a polarised epithelial cell line extensively used to study cilia-associated cell polarity. We generated mIMCD3 cells stably expressing doxycycline (dox) inducible NT (control), PRK1, PRK2 and PRK1+PRK2 shRNA (Supplemental Fig. 3A). In control cells 8–10 days after plating single cells, the growing spheroids begin to polarise, forming lumen-containing cysts (examples of lumen-cleared and non-cleared spheroids around 50 µm in diameter is shown in Fig. 3A). All cell lines were plated ±2 µg/ml dox for 10 days, after which the spheroid structures ≥50 µm were scored for having either cleared or non-cleared lumens. The ratio of cleared lumens ±dox (dox/no dox) was determined for each cell line and plotted (Fig. 3B). We found that depletion of either PRK1, PRK2 or both, ie. PRK1+PRK2 resulted in a decrease in the luminal clearance of spheroids, with PRK2 depletion showing the greatest significance (p = 0.011). There was no clear additive effect of depleting both PRK1 and PRK2. Thus, depletion of either PRK1, PRK2 or both, impairs spheroid lumen-formation similar to that seen upon depletion of other polarity-regulating transition zone proteins such as NPHP1, NPHP4 and NPHP8. Similar to depletion of PRK1/2, depletion of these transition zone proteins does not perturb cilia formation^31^.

The diameter of spheroids growing under the different conditions was determined as a function of selecting a cut off (50 µm) when scoring cleared and non-cleared structures (Fig. 3C and Supplemental Fig. 3B). A frequency plot of the different sizes of spheroids formed ±dox for each cell line was plotted to determine whether anchorage independent growth was affected (Fig. 3B). Where PRK2 was depleted by the presence of doxycycline (i.e. in PRK2 and PRK1+PRK2 cell lines), there was a significant reduction in spheroid diameter distribution (Fig. 3C). As before there was no additive effect of depleting both PRK1 and PRK2. Thus, whereas depletion of either PRK1 or PRK2 alone impairs establishment of cell polarity, only PRK2 depletion appeared to impair both polarity and reduce spheroid growth.

### Increased PRK2 expression is linked to poor survival in basal-like/triple negative breast cancer (TNBC) patients

**Figure 4:**
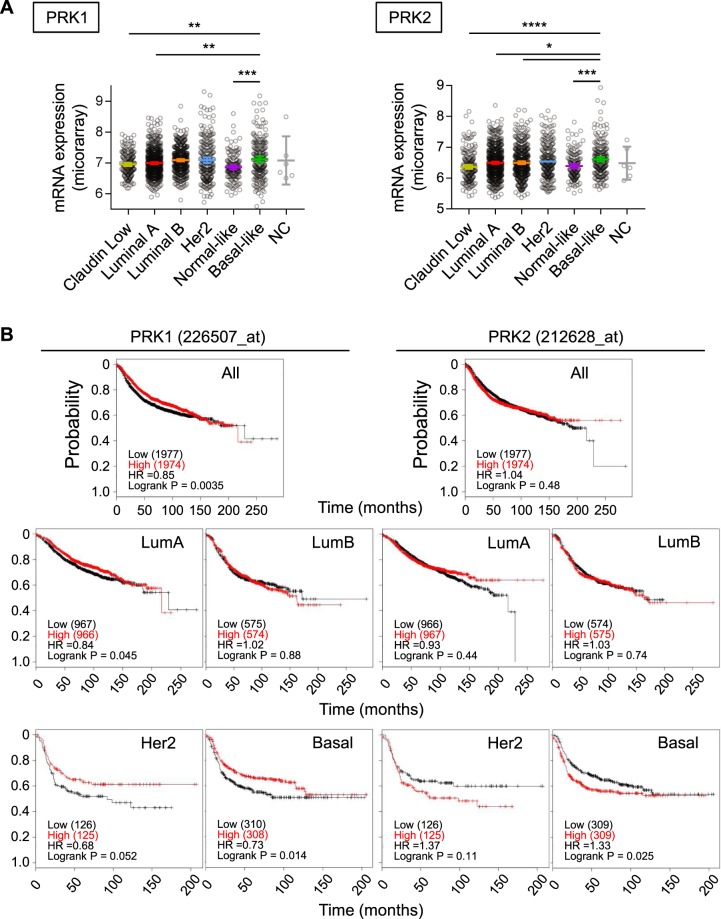
Increased PRK2 expression is linked to poor survival in basal-like/triple negative breast cancer (TNBC) patients. (**A**) Expression of PRK1 and PRK2 in breast cancer subtypes (METABRIC dataset accessed via cBioportal.org and plotted using Prism). (**B**) Kaplan Meier plots for PRK1 (left panel) and PRK2 (right panel) in all breast cancer patients and in specified subtypes.

The data presented above indicate a role for PRK1 and PRK2 in cell polarity and, in the case of PRK2, also in anchorage independent 3D spheroid growth. These traits are often dysregulated in cancers and interestingly it has been reported that amongst breast cancer subtypes Basal-like breast cancer cells retain the ability to form cilia^23,24^. We therefore next examined whether there was a link between PRK1 and PRK2 expression (cbioportal.org) and patient survival (kmplot.com^33^) in breast cancer (Fig. 4). The relative expression of PRK1 and PRK2 across the different breast cancer subtypes was plotted and shown in Fig. 4A. Expression of PRK1 and PRK2 in Basal-like tumours is significantly higher than in Normal-like, Luminal A and Claudin-low subtypes, while expression of PRK2 is also higher in Luminal B subtype. In terms of patient survival, higher PRK1 expression did not significantly correlate with poorer survival across all breast cancer patients or with a particular subtype (Fig. 4B, PRK1 panel); indeed, across all breast cancer patients and those with Basal-like and Her2 enriched tumours, higher PRK1 expression correlated with better survival. As with PRK1, PRK2 expression did not correlate with poorer survival across all breast cancer patients (Fig. 4B, PRK2 panel); however, there was a significant correlation (p = 0.025) between higher PRK2 expression and poor survival probability in patients with Basal-like/TNBC.

### Human and mouse TNBC cells are more sensitive to PRK2 depletion

**Figure 5:**
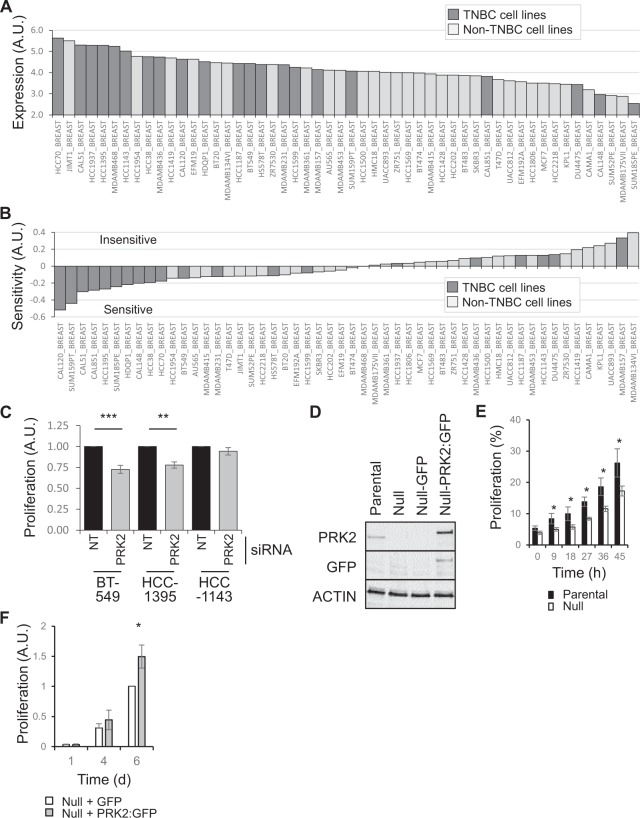
Human and mouse TNBC cells are more sensitive to PRK2 depletion. (**A**) PRK2 expression across a panel of breast cancer cell lines (Depmap.org). (**B**) Sensitivity to PRK2 depletion in a panel of breast cancer cell lines (Depmap.org). (**C**) Relative proliferation of 3 TNBC cell lines upon siRNA induced PRK2 depletion. Non-targeting (NT) siRNA oligos used as control. (**D**) Western blot analysis confirming deletion of PRK2 in MET1 cells and the subsequent exogenous expression of PRK2 fused to GFP (PRK2:GFP). (**E**) Relative proliferation of MET1 (Parental) and MET1 cells null for PRK2 (Null). (**F**) Relative proliferation of Null cells expressing GFP or PRK2:GFP. Unless stated otherwise, n ≥ 3 (±s.e.m.).

The data presented above (mIMCD3 spheroid growth assays (Fig. 3C), PRK1 and PRK2 expression in breast cancer subtypes (Fig. 4A) and the poor patient survival correlation with high PRK2 expression (Fig. 4B) all indicate that PRK2 plays an important role in cancer biology. The data however say nothing of how patients, or indeed cell lines, are likely to respond to the targeting of PRK2 activity. Thus to gain further insight into this we looked at the expression of PRK2 across BC cell lines and their sensitivity to PRK2 depletion (Fig. 5A,B). Using the Depmap database (depmap.org) we find that TNBC cell lines tend to have higher expression of PRK2 (Fig. 5A) and are also more sensitive to its depletion (Fig. 5B). For confirmation we selected three cell lines (two sensitive (BT-549 and HCC-1395) and one insensitive (HCC-1143)) and carried out siRNA mediated depletion of PRK2 (Fig. 5C). In agreement with the Depmap data the proliferation of BT-549 and HCC-1395 cells was reduced while that of HCC-1143 cells was unaffected. siRNA mediated depletion of PRK2 was confirmed in HCC-1143 and BT-549 cells (Supplemental Fig. 4A).

The generation of genetic tools to study novel targets for therapeutic intervention is critical for the understanding of both the underlying mechanisms of action through basic cell biology studies and for more complex *in vivo* studies. We thus deleted PRK2 using CRISPR/Cas9 technology in a mouse TNBC cell line, MET1^34^. These cells are not only amenable to both basic cell biology and *in vivo* studies but also offer a syngeneic model allowing for the possibility to study the tumour:host immune interplay. We confirmed deletion and reintroduction of PRK2 fused to GFP (PRK2:GFP) using western blot analysis (Fig. 5D). As with the sensitive human TNBC cell lines, loss of PRK2 in MET1 cells also led to reduced proliferation (Fig. 5E) and, importantly, exogenous expression of PRK2:GFP rescued this (Fig. 5F).

### Commonalities between ciliating and non-ciliating (cancer) cells

In order to determine the commonalities between ciliating and non-ciliating cancer cells we first looked to see if the two key observations from ciliating cells, i.e. increased pPRK1/pPRK2 levels upon serum withdrawal and reduced spheroid growth upon PRK1 and/or PRK2 depletion, are also observed in TNBC MET1 cells. In breast cancer, though cilia formation is thought to be lost early in its development^23^, expression of cilia associated proteins persist and in some cases contribute to cancer development/progression. Therefore we then identified the binding partners of PRK2 in MET1 cells and looked for the presence of proteins that are linked to both cilia formation and TNBC.

**Figure 6:**
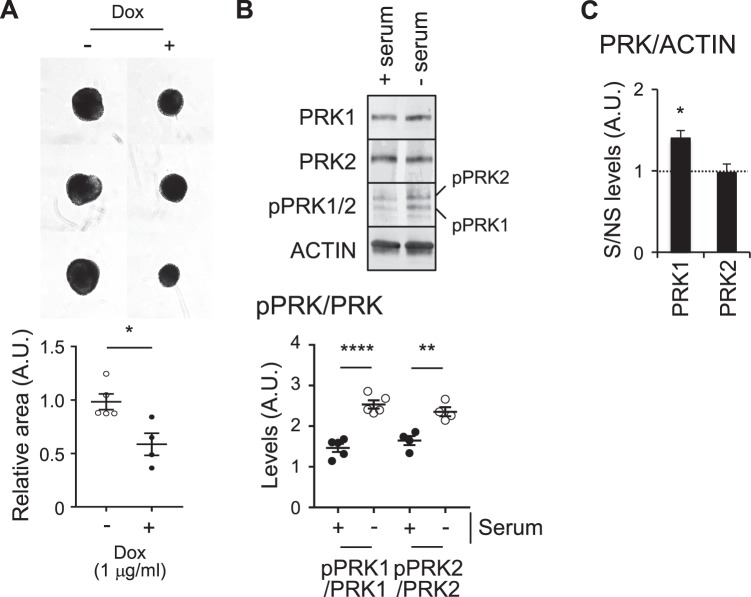
Commonalities between ciliating and non-ciliating TNBC cancer cells. (**A**) Spheroids formed by mouse TNBC MET1 depleted of PRK1 + PRK2 using doxycycline (dox) inducible shRNA (images, top and quantification, below). (**B**) Western blot analysis (top) and quantification (below) of lysate from cells ± serum. (**C**) Quantification of total PRK1 levels (± serum) by western blot analysis. Unless stated otherwise n ≥ 3 (±s.e.m.).

As seen with cilia forming cells, doxycycline induced shRNA co-depletion of PRK1 and PRK2 resulted in decreased spheroid growth (~40%) relative to control cells (Fig. 6A), while serum withdrawal resulted in increased pPRK1/pPRK2 levels (Fig. 6B). In addition, there was also a significant increase in total PRK1 levels (but not PRK2 levels) upon serum withdrawal (Fig. 6C). We next made use of the PRK2 null-GFP and PRK2 null-PRK2:GFP cells to determine the interactome of PRK2 in MET1 cells (Supplement Fig. 5A). To help visualise the connectivity between PRK2 interactors and them to cilia and breast cancer, we combined all of the known PRK2 interactors with our dataset (Supplemental Fig. 4A) and inputted it into string-db.org (Supplemental Fig. 5B). Using targetvalidation.org we found that a number of PRK2 interactors are known be linked to cilia biology (EEF1A1, FLNA, TCP1, CCT4, CCT2 EHD1, MLKL and GSK3B). All of these, with the exception of TCP1, have also been linked to breast cancer, while FLNA, GSK3B and CCT2 are specifically linked to TNBC. In addition EEF1A1, which is present in both our dataset and in thebiogrid.org dataset is linked to both cilia and breast cancer biology (Supplemental Fig. 5A,B).

## Discussion

Here we present evidence that PRK1 and PRK2 play a role in cilia biology and contribute to cancer-associated phenotypes such as polarity and 3D spheroid growth – processes that are important in both cilia and cancer biology. Their increased phosphorylation upon serum withdrawal in both cilia forming non-cancer cells and cancer cells suggests a role for PRKs in sensing extracellular cues and modulating, but not defining responses. Finally, we present evidence linking PRK2 to basal-like/triple negative breast cancer (TNBC) and have shown that depletion of PRK2 in TNBC cell lines impairs proliferation and 3D spheroid growth. The link between PRK2 and breast cancer is in itself important as it reveals a possible new avenue for targeting TNBC, which is a highly aggressive cancer subtype with poor outcome for patients.

PRK1 and PRK2 are two closely related proteins belonging to the AGC family of kinases that are thought to regulate the actin cytoskeleton^1,11,20^. Their activation results from phosphorylation by PDPK1 (after synthesis), auto-phosphorylation and by the binding of small Rho family GTPases and lipids^35^. Here we have shown that phosphorylated PRK1 and PRK2 (pPRK1/pPRK2) localise to the transition zone of cilia and that their depletion impairs PDGF and Hedgehog signalling, modulates cilia length and planer polarity. There is abundant evidence that transition zone proteins exist in complexes that regulate entry and exit of proteins into the cilium and that this is at least in part through the regulation of the actin cytoskeleton^31,36,37^. Therefore, PRK protein activities are candidate regulators, or participants, in such complexes as they are localised at the place to do so and their inherent ability to regulate the actin cytoskeleton. Of interest, another AGC family kinase, PRKACA, also localises to the base of cilia and regulates hedgehog signalling^38^.

A panoply of diseases together termed ciliopathies have been attributed to the loss of specific cilia associated proteins. PRK2 has recently been linked to a newly established ciliopathy, STAR syndrome, where loss of Cdk10/ClnM activity in humans results in developmental abnormalities such as toe syndactyly, telecanthus, and anogenital and renal malformations^25,26^. Cdk10/ClnM is suggested to phosphorylate PRK2 to facilitate PRK2-RHO association (thus both enhancing PRK2 activity and conferring RHO stabilization upon serum withdrawal), to modulate cilia through regulation of the actin cytoskeleton. Our novel observation that phosphorylated PRK2 (and PRK1) localises to the transition zone of cilia consolidates its role as a bon-fide cilia associated protein and furthermore broadly supports a role for PRK-RHO regulation of the actin cytoskeleton in ensuring normal cilia function. Finally, this study provides a functional and mechanistic insight into the role of PRKs at cilia.

Another possible link between PRKs and cilia is through a PRK2 interacting protein, Cep19 (thebiogrid.org), whose loss in humans and in mice results in morbid obesity^39^. Mechanistically Cep19 is responsible for localizing proteins (such as the RABL2B GTPase complex) to the base of cilia^40^. Though deletion of PRK2 in mouse is embryonic lethal^13^ the authors report that the PRK2 null embryonic fibroblasts (MEFs) showed impaired migration – a phenotype which can result from impaired cilia function. PRK1 has also been targeted for deletion in mouse and though it is not lethal (www.informatics.jax.org/marker/phenotypes/MGI:108022), the mice have a variety of phenotypes including increased body fat, which, is intriguingly similar to the obesity phenotype seen with CEP19 deletion. As evidenced by the *in vivo* PRK1 and PRK2 deletion phenotypes, though PRK1 cannot compensate for PRK2 during development, their highly similar nature may allow for a partial level of redundancy.

Relatively little is known about the roles of PRK1 and PRK2 in cancer and in particular BC. Our data show that PRK2 regulates proliferation, planer polarity and spheroid growth, which are all key cancer associated phenotypes. We also show that TNBC cell lines tend to have higher PRK2 expression and are more likely to be sensitive to its loss. Finally though patients with TNBC have significantly higher expression of both PRK1 and PRK2, only higher expression of PRK2 is also associated with a poorer survival outcome. The combination of the cell line and patient data suggest that PRK2 is potentially a novel therapeutic target in BC and more specifically in TNBC – a patient cohort that has poor clinical outcome.

In terms of cilia and BC development, there is evidence that cilia are lost early during cancer development^23,24^ and in agreement with this we have not observed cilia in the TNBC cell line MET1. However we cannot categorically rule out their presence at a very low incidence. In both studies that investigated the role of cilia in BC, Basal-like cells were identified as being the only subtype that retained the ability (though at very low incidence) to form cilia. Basal-like tumours have generally higher expression of PRK2, which correlates with poorer outcome, while TNBC cell lines express PRK2 at higher levels and are more sensitive to its depletion. Finally, Menzl *et al*. go on to speculate that these low incidence ciliated Basal-like cells have mal/poorly functioning cilia and moreover, that they represent a chemo-resistant subpopulation. We have also shown that depletion of PRK1 and PRK2 disrupts normal cilia associated signalling. Taken together this is suggestive of a more direct link between PRK1/PRK2, cilia and BC, where PRK1/PRK2 potentially regulate the signalling from the poorly/mal functioning cilia and contribute to cancer associated phenotypes. Confirming if such a direct link exists/operates will require a far more protracted and in depth study and is beyond the scope of this current study. Furthermore to fully realise the utility of targeting PRK2 and/or PRK1 in TNBC, the links between PRK1 and PRK2 in cilia formation and signalling in the context of the chemo-resistance subpopulation should also be addressed.

As mentioned, though the ability to form cilia may be lost early during cancer development, many of the components required to form and signal from cilia remain present and contribute to cellular signalling and to cancer development and progression^41^. In MET1 cells PRK2 (which our data suggests plays a more prominent role in BC biology phenotypes than PRK1 does) co-immunoprecipitates with a number of proteins (FLNA, GSK3B and CCT2) that are functionally linked to both cilia biology and TNBC (targetvalidation.org). The regulation of these and other proteins by PRK2 may contribute to TNBC and as such small molecule kinase inhibitors of PRK2 may have therapeutic potential. Moreover such inhibitors will also greatly aid in determining the role of PRK2 in cilia biology and whether PRK2 related ciliary phenotypes contribute/drive BC phenotypes.

Carrying out *in vivo* studies in a relevant background and within the context of drug resistance and advanced metastatic disease will be of particular importance in further defining the role of PRK2 as a potential therapeutic target and thus help inform the development of novel small-molecule PRK inhibitors. In the absence of such selective small molecule inhibitors, which target PRK1 and PRK2, our current study applies the latest advances in functional genomics, phenotypic characterization and interactome analysis to elucidate the biological roles of PRK1 and PRK2 at cellular and pathway levels. This work has clearly outlined a role for PRK2 in the basal-like/TNBC setting.

## Materials and Methods

### Cell culture and treatments

All cell lines were purchased from ATCC and cultured in DMEM containing glutamine and 10% FBS under standard conditions (humidity, 37 °C and 5% CO_2_) unless stated otherwise. Mouse IMCD3 cells were cultured in DMEM F-12 medium plus 10% FBS. For serum starvation, cells were washed 3 times in PBS and bathed in DMEM containing glutamine for overnight 16–20 hours (h) under standard conditions. For PDGF-AA (R&D Systems, 221-AA-010) stimulation, serum starved cells were treated with DMSO or 50 ng/ml of PDGF-AA for 0, 3 and 10 minutes (min). For serum stimulation, serum starved cells were treated with DMSO or 50 ng/ml of PDGF-AA for 0, 3 and 10 minutes (min). For purmorphamine (Sigma, SML0868) stimulation, serum starved cells were treated with DMSO or 5 µM purmorphamine for 0, 3 and 6 h.

### Transfections

For siRNA transfections, cells were transfected using Lipofectamine 2000 RNAmax (according to manufacturers’ (Invitrogen) recommendation) + siRNA oligos (20–80 nM) followed by media exchange the following day. Cells were harvest for western blot or immunofluorescence analysis after 48 h post transfection. For generating stable and doxycycline inducible shRNA cell lines, HEK293T cells were transfected with the relevant plasmid and after 48 h the media was used to infect cells. 48 h after infection, cells were treated with 2 µg/ml doxycycline for 16 h and the fluorescent cell isolated using FACS sorting. 48 h post sorting, cells were selected using the appropriate selection.

### Planer polarity and 3D spheroid assays

mIMCD3 cells (Planer polarity) were seeded as single cells in 2% Geltrex (Gibco, A14132–02) in standard media ±2 mg/ml doxycycline and fixed (10% formaldehyde in PBS for 20 min) and washed on day (d) 8. Structures were blocked (1% BSA in PBS) and stained with DAPI and TRITC phalloidin and imaged using 20x objective. 2000 MET1 cells (3D spheroid) were plated per well into U-bottomed low adherence 96-well plates (Corning, 7007), centrifuged (800 g for 5 min) and imaged at d 2 and at d 8.

### Western blot and immunofluorescence

For western blots, at the endpoint of experiments the cells were washed in ice cold PBS and lysed using standard RIPA buffer containing protease and phosphatase inhibitors (Roche, 11836145001 and 04906837001, respectively). Samples (20 µg of protein) were run out on a 4–15% gradient gel (BioRad, 4561085), transferred, blocked (5% BSA) and probed with the appropriate primary antibodies (1/1000) over night at 4 °C. Secondary antibodies were probed as recommended by the manufacturer (LI-COR). For immunofluorescence, at the endpoint of experiments the cells on coverslips were fixed (3.7% formaldehyde in PBS + 0.1% Triton X-100), washed, blocked (2% BSA) and probed with the appropriate primary antibodies (1/100) over night at 4 °C. Secondary antibodies (1/200) were probed at room temperature for 1 h washed off and mounted using vectashield containing DAPI. Intensity of bands were quantified using ImageJ/Fiji software.

### Imaging

Cells on coverslips and mIMCD3 structures in flat-bottomed tissue culture plates were imaged using a standard confocal microscope (Olympus FV1000). Intensity of regions were quantified using ImageJ/Fiji software. Spheroids growth in U-bottomed plates were imaged on the ImageXpress Micro XLS (Molecular Devices) at 4x magnification and analysis of spheroid size performed using the MetaXpress image analysis software. Live cell imaging (IncuCyte imaging system) was used to automatically determine the proliferation of cells by applying a confluence algorithm mask measuring the imaged area covered by proliferating cells over time.

### Antibodies

Abcam; PKN1 (195264): Millipore; PKN2 (MABT496): CST; pPRK1/pPRK2 (2611), Actin (3700), Akt (4051), p(308)Akt (2965), p(473)Akt (4060), ERK (4696), pERK (4370), Pdpk1 (3062): LI-COR; IRDye 800CW goat anti-rabbit (P/N 925–32210), IRDye 680LT goat anti-mouse IgG (P/N 925–68020): Invitrogen (Alexa Fluor); goat anti-mouse 488 (A32723), goat anti-mouse 594 (A11032), goat anti-rabbit 488 (A11034), goat anti-rabbit 594 (R37117): LifeSpan BioSciences; FITC conjugated NPHP1 (LS-C317167).

### si/shRNA sequences

All siRNA oligos were purchased from Dharmacon; non-targeting (NT) (D-001810–10–05), PRK1 (0J-065212–05–0002 and 0J-065212-07-0002) and PRK2 (0J-0655210-05-0002 and 0J-065210-07-0002). Doxycycline inducible shRNA constructs were purchased from Origene; NT (RHS4743), PRK1 (V3SM11253-231861862) and PRK2 (V3SM11253-231040202).

### Mass-spec analysis

Cells were seeded for 24 h, serum starved overnight and lysed (RIPA buffer containing protease and phosphatase inhibitors (Roche)) after 2 washes in ice-cold PBS. 500 µg of protein was processed for GFP immunoprecipitation and protein digestion in Kingfisher Duo Robot (briefly, samples were incubated with 5 µl of GFP-Trap-MA beads (Chromotek) for 1 h at 4 C, followed by 2 washes with RIPA buffer and 3 washes of PBS buffer). Proteins were eluted and digested in UA buffer (2 M Urea, 50 mM Tris (pH 7.5), 1 mM DTT) containing 0.5 µg of trypsin (Promega) for 6 hours at 37 C. Peptides were then desalted and injected in liquid chromatograpy-tandem MS as described previously^42^. MaxQuant software was used to search for peptides and to calculate label-free intensities of the corresponding proteins. Specific interactors were determined from three biological replicates, as the proteins that were enriched at least two fold (and p = <0.05) in PRK2 expressing cells when compared with corresponding GFP-empty vector.

### Statistical analysis

Students T-tests were performed where significance tests were carried out – unless stated otherwise. Significance of the asterisk(s) are as follows:- * represents <0.05, ** represents <0.01, *** represents <0.001 and **** represents <0.0001.

Figure 2A – significance was tested between the ratios of pPRK1/PRK1 or pRRK2/PRK2 ± serum.

Figure 2C – significance was tested in the difference of length between cilia of NT and PRK1 + PRK2 siRNA treated cells.

Figure 2D – significance was tested in the ratios of pAKT (308)/AKT, pAKT (473)/AKT and pERK/ERK at 10 minutes post stimulation between NT and PRK1 or PRK2 siRNA treated cells.

Figure 2E – significance was tested in the ratios of pAKT (308)/AKT and pAKT (473)/AKT between NT and PRK1 + PRK2 siRNA treated cells at 0, 3 and 10 minutes post stimulation.

Figure 2F – significance was tested in the intensity of GLI3 staining at the tips of cilia in NT and PRK1 + PRK2 siRNA treated cells.

Figure 3B – significance was tested for the formation of cleared cysts in doxycycline (dox) inducible cells where dox induces the expression of NT, PRK1, PRK2 or PRK1 + PRK2 shRNA.

Figure 3C – significance was tested for the difference in size of spheroids/cycts in doxycycline (dox) inducible cells where dox induces the expression of NT, PRK1, PRK2 or PRK1 + PRK2 shRNA.

Figure 4A – significance was tested in the expression of PRK1 or PRK2 between Basal-like subtype and others individually. 1way ANOVA, multiple comparisons option was used in Prism.

Figure 5C – significance was tested between the difference in proliferation of NT and PRK2 siRNA treated cells after 5 days.

Figure 5E – significance was tested between the proliferation of MET1 parental and MET1-PRK2 null cells.

Figure 5F – significance was tested between the proliferation of MET1-PRK2 null cells expressing either GFP or PRK2 fused to GFP cells at day 1, 4 and 6.

Figure 6A – significance was tested in the size of spheroids MET1 cells ± dox, where the presence of dox leads to depletion of PRK1 and PRK2.

Figure 6B – significance was tested between the ratios of pPRK1/PRK1 or pRRK2 / PRK2 ± serum.

Figure 6C – significance was tested between the ratios of PRK1/Actin ± serum.

## Supplementary information

Supplementary Data.

## Data Availability

All of the data supporting this study is provided in the main article and the supplementary information accompanying this paper.

## References

[1] Dong LQ (2000). Phosphorylation of protein kinase N by phosphoinositide-dependent protein kinase-1 mediates insulin signals to the actin cytoskeleton. Proc. Natl. Acad. Sci. USA.

[2] Mukai H, Ono Y (1994). A Novel Protein-Kinase With Leucine Zipper-Like Sequences - Its Catalytic Domain Is Highly Homologous to That of Protein- Kinase-C. Biochemical biophysical Res. Commun..

[3] Flynn P, Mellor H, Casamassima A, Parker PJ (2000). Rho GTPase control of protein kinase C-related protein kinase activation by 3-phosphoinositide-dependent protein kinase. J. Biol. Chem..

[4] Vincent S, Settleman J (1997). The PRK2 kinase is a potential effector target of both Rho and Rac GTPases and regulates actin cytoskeletal organization. Mol. Cell. Biol..

[5] Amano M (1996). Identification of a putative target for Rho as the serine-threonine kinase protein kinase N. Sci..

[6] Palmer RH, Ridden J, Parker PJ (1994). Identification of multiple, novel, protein kinase C-related gene products. FEBS Lett..

[7] Palmer R, Dekker L, Woscholski R, Good J, Gigg R (1995). Activation of PRK1 by phosphatidylinositol 4, 5-bisphosphate and phosphatidylinositol 3, 4, 5-trisphosphate. J. Biol. Chem..

[8] Morrice NA, Fecondo J (1994). & Wettenhall, hard E. H. Differential effects of fatty acid and phosholipid activators on the catalytic activities of a structurally novel protein kinase from rat liver. FEBS Lett..

[9] Standaert M (1998). Comparative Effects of GTP g S and Insulin on the Activation of Rho, Phosphatidylinositol 3-Kinase, and Protein Kinase N in Rat Adipocytes. J. Biol. Chem..

[10] Takahashi M, Mukai H, Toshimori M, Miyamoto M, Ono Y (1998). Proteolytic activation of PKN by caspase-3 or related protease during apoptosis. Proc. Natl. Acad. Sci..

[11] Lachmann Sylvie, Jevons Amy, De Rycker Manu, Casamassima Adele, Radtke Simone, Collazos Alejandra, Parker Peter J. (2011). Regulatory Domain Selectivity in the Cell-Type Specific PKN-Dependence of Cell Migration. PLoS ONE.

[12] Misaki K (2001). PKN delays mitotic timing by inhibition of Cdc25C: possible involvement of PKN in the regulation of cell division. Proc. Natl. Acad. Sci. USA.

[13] Quétier I (2016). Knockout of the PKN Family of Rho Effector Kinases Reveals a Non-redundant Role for PKN2 in Developmental Mesoderm Expansion. Cell Rep..

[14] Matsuzawa K (1997). Domain-Specific Phosphorylation of Vimentin and Glial Fibrillary Acidic Protein by PKN. Biochem. Biophys. Res. Commun..

[15] Taniguchi T (2001). Phosphorylation of Tau Is Regulated by PKN. J. Biol. Chem..

[16] Metzger E (2008). Phosphorylation of histone H3 at threonine 11 establishes a novel chromatin mark for transcriptional regulation. Nat. Cell Biol..

[17] Harrison BC (2010). Protein kinase C-related kinase targets nuclear localization signals in a subset of class IIa histone deacetylases. FEBS Lett..

[18] Chang S, Bezprozvannaya S, Li S, Olson EN (2005). An expression screen reveals modulators of class II histone deacetylase phosphorylation. Proc. Natl. Acad. Sci. USA.

[19] Bourguignon LYW, Gilad E, Peyrollier K, Brightman A, Swanson RA (2007). Hyaluronan-CD44 interaction stimulates Rac1 signaling and PKNγ kinase activation leading to cytoskeleton function and cell migration in astrocytes. J. Neurochem..

[20] Watanabe G (1996). Protein kinase N (PKN) and PKN-related protein rhophilin as targets of small GTPase Rho. Sci..

[21] Ciência IGD (2012). Centrosomes and Cilia in Human Disease. Trends Genet..

[22] Higgins, M., Obaidi, I. & McMorrow, T. Primary cilia and their role in cancer (Review). Oncology Letters. 10.3892/ol.2019.9942 (2019). 10.3892/ol.2019.9942 PMC6396132 30867732

[23] Menzl Ina, Lebeau Lauren, Pandey Ritu, Hassounah Nadia B, Li Frank W, Nagle Ray, Weihs Karen, McDermott Kimberly M (2014). Loss of primary cilia occurs early in breast cancer development. Cilia.

[24] Yuan Kun, Frolova Natalya, Xie Yi, Wang Dezhi, Cook Leah, Kwon Yeon-Jin, Steg Adam D., Serra Rosa, Frost Andra R. (2010). Primary Cilia Are Decreased in Breast Cancer: Analysis of a Collection of Human Breast Cancer Cell Lines and Tissues. Journal of Histochemistry & Cytochemistry.

[25] Guen VJ (2016). STAR syndrome-associated CDK10/Cyclin M regulates actin network architecture and ciliogenesis. Cell Cycle.

[26] Guen VJ (2013). CDK10/cyclin M is a protein kinase that controls ETS2 degradation and is deficient in STAR syndrome. Proc. Natl. Acad. Sci..

[27] Fliegauf Manfred, Horvath Judit, von Schnakenburg Christian, Olbrich Heike, Müller Dominik, Thumfart Julia, Schermer Bernhard, Pazour Gregory J., Neumann Hartmut P.H., Zentgraf Hanswalter, Benzing Thomas, Omran Heymut (2006). Nephrocystin Specifically Localizes to the Transition Zone of Renal and Respiratory Cilia and Photoreceptor Connecting Cilia. Journal of the American Society of Nephrology.

[28] Schneider L (2005). PDGFRαα signaling is regulated through the primary cilium in fibroblasts. Curr. Biol..

[29] Corbit KC (2005). Vertebrate Smoothened functions at the primary cilium. Nat..

[30] Wen X (2010). Kinetics of Hedgehog-Dependent Full-Length Gli3 Accumulation in Primary Cilia and Subsequent Degradation. Mol. Cell. Biol..

[31] Sang, L. et al. Mapping the Nephronophthisis-Joubert-Meckel-Gruber Protein Network Reveals Ciliopathy Disease Genes and Pathways. 145, 513–528 (2012). 10.1016/j.cell.2011.04.019 PMC3383065 21565611

[32] Wallingford JB, Mitchell B (2011). Strange as it may seem: the many links between Wnt-signaling, planar cell polarity, and cilia. Genes. Dev..

[33] Györffy Balazs, Lanczky Andras, Eklund Aron C., Denkert Carsten, Budczies Jan, Li Qiyuan, Szallasi Zoltan (2009). An online survival analysis tool to rapidly assess the effect of 22,277 genes on breast cancer prognosis using microarray data of 1,809 patients. Breast Cancer Research and Treatment.

[34] Borowsky Alexander D., Namba Ruria, Young Lawrence J.T., Hunter Kent W., Hodgson J. Graeme, Tepper Clifford G., McGoldrick Erik T., Muller William J., Cardiff Robert D., Gregg Jeffrey P. (2005). Syngeneic mouse mammary carcinoma cell lines: Two closely related cell lines with divergent metastatic behavior. Clinical & Experimental Metastasis.

[35] Leroux Alejandro E., Schulze Jörg O., Biondi Ricardo M. (2018). AGC kinases, mechanisms of regulation ‎and innovative drug development. Seminars in Cancer Biology.

[36] Garcia-gonzalo, F. R. et al. A Transition Zone Complex Regulates Mammalian Ciliogenesis and Ciliary Membrane Composition Francesc. 43, 776–784 (2012). 10.1038/ng.891 PMC3145011 21725307

[37] Francis SS, Sfakianos J, Lo B, Mellman I (2011). A hierarchy of signals regulates entry of membrane proteins into the ciliary membrane domain in epithelial cells. J. Cell Biol..

[38] Barzi M, Berenguer J, Menendez A, Alvarez-Rodriguez R, Pons S (2010). Sonic-hedgehog-mediated proliferation requires the localization of PKA to the cilium base. J. Cell Sci..

[39] Shalata A (2013). Morbid obesity resulting from inactivation of the ciliary protein CEP19 in humans and mice. Am. J. Hum. Genet..

[40] Kanie T (2017). The CEP19-RABL2 GTPase Complex Binds IFT-B to Initiate Intraflagellar Transport at the Ciliary Base. Dev. Cell.

[41] Tamina Seeger-Nukpezah, J L. L, Victoria Serzhanova, and E. A. G. Cilia and cilia-associated proteins in cancer. 10, 135–142 (2013). 10.1016/j.ddmec.2013.03.004 PMC4073101 24982684

[42] Turriziani Benedetta, von Kriegsheim Alexander, Pennington Stephen R. (2016). Protein-Protein Interaction Detection Via Mass Spectrometry-Based Proteomics. Modern Proteomics – Sample Preparation, Analysis and Practical Applications.

