# Supplementary material for: Novel roles of PRK1 and PRK2 in cilia and cancer biology
Source: Sci Rep. 2020 Mar 3;10:3902. doi: 10.1038/s41598-020-60604-3 (PMC7054267; doi:10.1038/s41598-020-60604-3)
Supplement: Supplementary file 1 — Supplementary Data. [file 41598_2020_60604_MOESM1_ESM.pdf]

Novel roles of PRK1 and PRK2 in cilia and cancer biology

Hitesh Patel*,2,1, Jun Li1, Ana Herrero1, Jakob Kroboth1, Adam Byron1, Alex Von Kriegsheim1, Valerie Brunton1, Neil Carragher1, Toby Hurd*,1 and Margaret Frame1

Correspondence (*) to hp264@sussex.ac.uk
1 University of Edinburgh, Edinburgh Cancer Research UK Centre, Institute of Genetics and Molecular Medicine, Crewe Road South
2 University of Sussex, Sussex Drug Discovery Centre, School of Life Sciences, Brighton, BN1 9QJ

Supplemental Figure 1

A

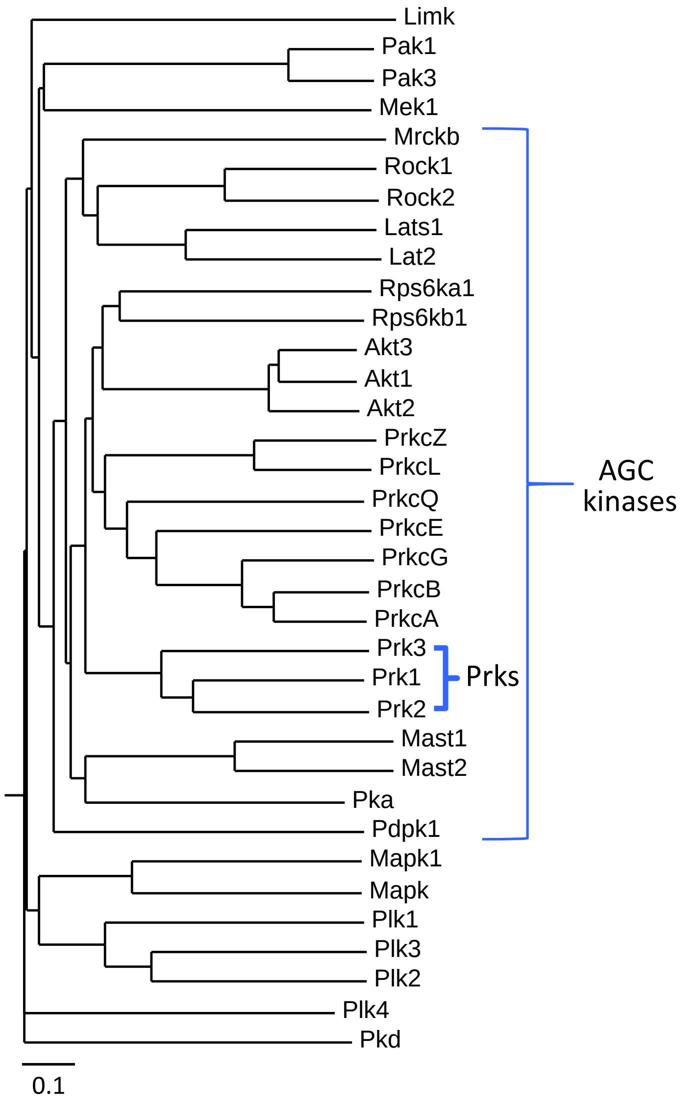

C

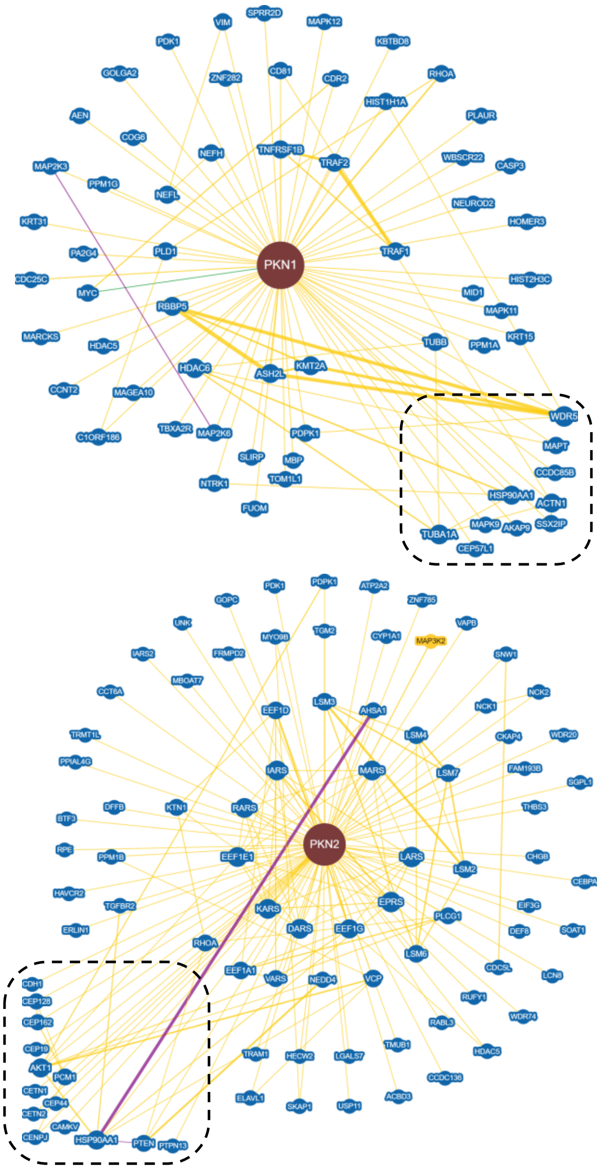

B

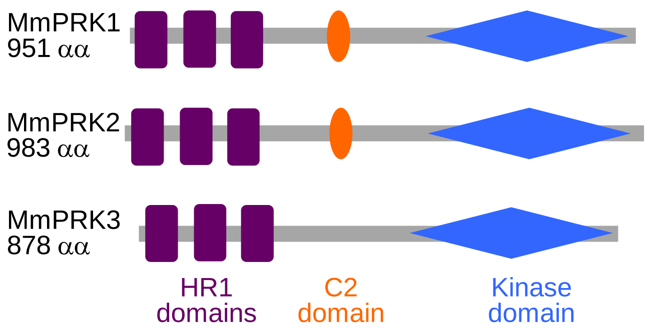

Sequence identity (%)			
	PRK3	PRK2	PRK1
PRK3	100	52.93	53.14
PRK2	52.93	100	61.04
PRK1	53.14	61.04	100

D

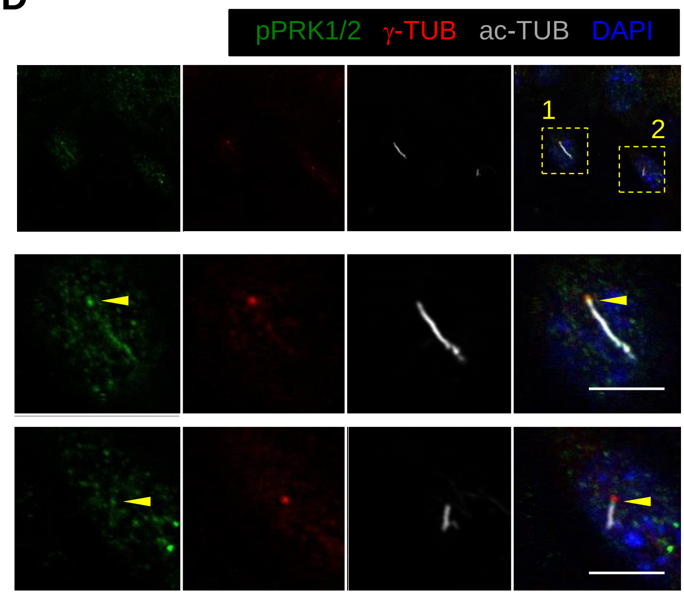

Supplemental Figure 2

A

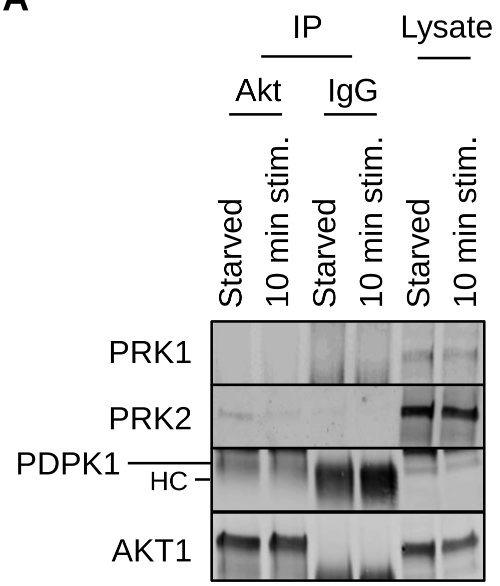

Supplemental Figure 3

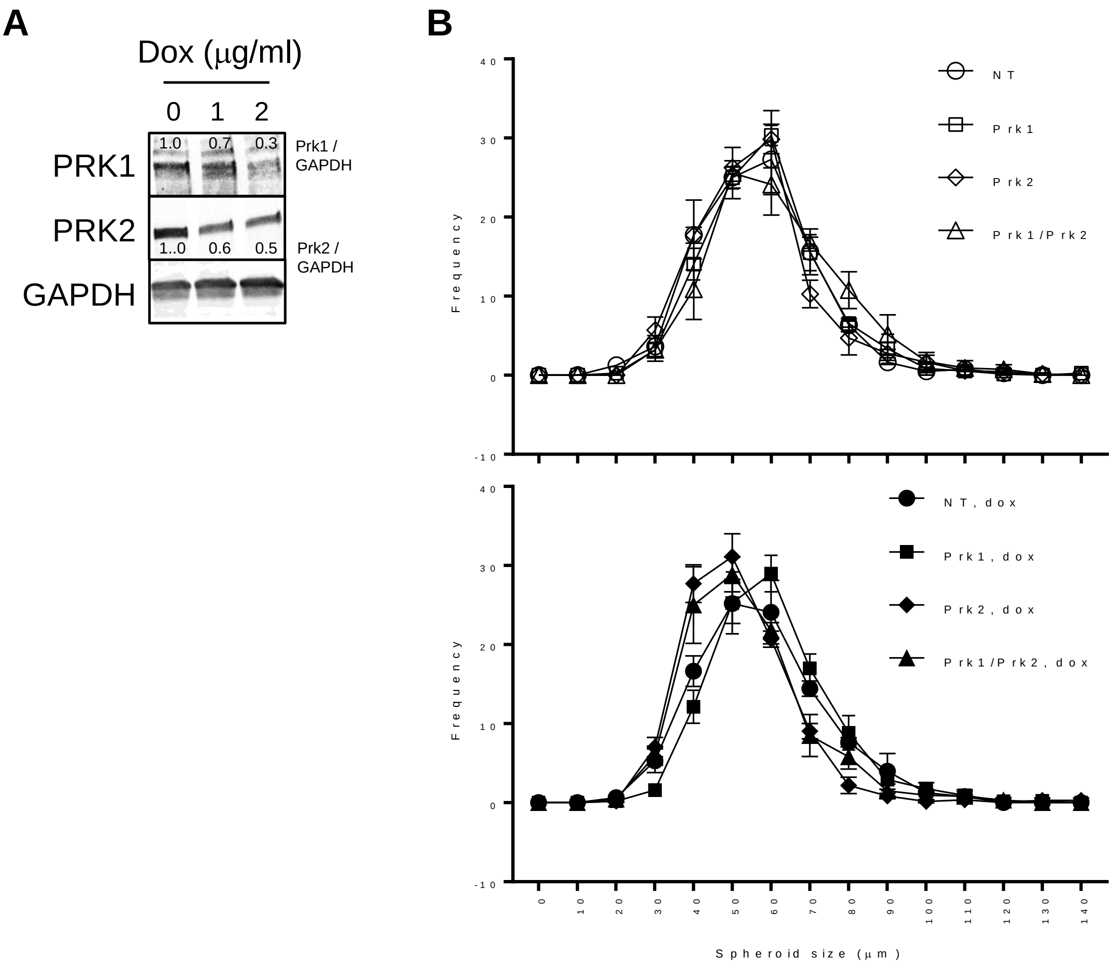

Supplemental Figure 4

A

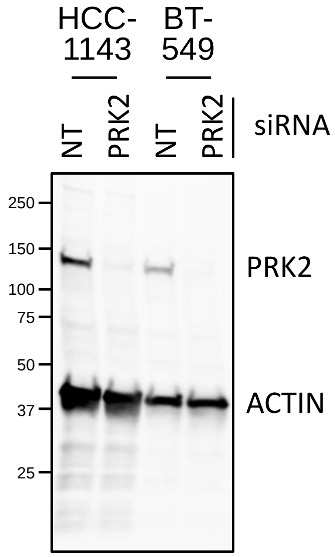

Supplemental Figure 5

A

PKN2	NCK1	PLCG1	RHOA	PDPK1	PDK1	AKT1	PTPN13	EEF1E1	TRMT1L	NCK2	MAP3K2	USP11	WDR20	HDAC5	PTEN	NEDD4	CEBPA	ELAVL1	VCP	HSP90AA1
HECW2	FRMPD2	TGFBR2	SKAP1	THBS3	CYP1A1	UNK	CCT6A	PCM1	CEP162	CEP128	CEP44	CEP19	CETN2	CENPJ	RUFY1	SNW1	CDC5L	AHSA1	CDH1	CETN1
CAMKV	RABL3	VAPB	GOPC	ZNF785	DFFB	TMUB1	DEF8	HAVCR2	PPIAL4G	LGALS7	CKAP4	TRAM1	LARS	WDR74	MBOAT7	ERLIN1	CCDC136	CHGB	MYO9B	FAM193B
EIF3G	EEF1A1	ACBD3	LSM2	LSM3	LSM4	LSM6	LSM7	PPM1B	RPE	ATP2A2	BTF3	DARS	EEF1D	EEF1G	EPRS	IARS	KARS	KTN1	MARS	RARS
SOAT1	VARS	SGPL1		VIM	HNRNPA3	SNRPN	MLKL	RNPS1	CSNK1D	UMPS	DDX20	UBA52	CCT4	EEF1A1	HSPA8	PKM	RPS6	RPS11	RPS3	CFL1
LMNA	EIF4A1	RPL23A	RPL15	HSP90AB1	RPS2	RPL31	PLEC	RPS14	RPS12	RPL7A	PHB2	FLNA	RPL26	RPS27	RPL10	HSPA1B	DDX5	FASN	SURF6	RPS24
HNRNPM	HNRNPH1	ALDH18A1	GVIN1	RPL27	PHB	TCP1	RPS7	MAGI3	RPS26	RPL9	ENO1	RPS9	PHGDH	DX39A	RPS16	RPL21	YWHAG	MCM6	HNRN	HSPD1
GEMIN5	RPL5	GSK3B	THBS1	MYL6	RPL34	DSTN	PDCD6IP	RHEB	ANXA1	SGSM2	SLC25A3	HNRNPA1	CCT6B	ALDH2	MCCC1	SNRPA	HIST1H1C	SRSF7	SFXN3	RPL35A
HNRNPK	BLVRA	NUP93	FUBP3	FARP1	CXORF56	FAR1	MAT2A	MYOF	SNRNP70	RPL39	MAP2K2	NAT10	CCT2	ACP1	SMARCA1	EHD1	PTPN11	PUS1	LRRC40	RPS18
USP3		PKN1	RHOB	RHOC	RAC1	PKN3	CDC25C	PPP1R14A												

B

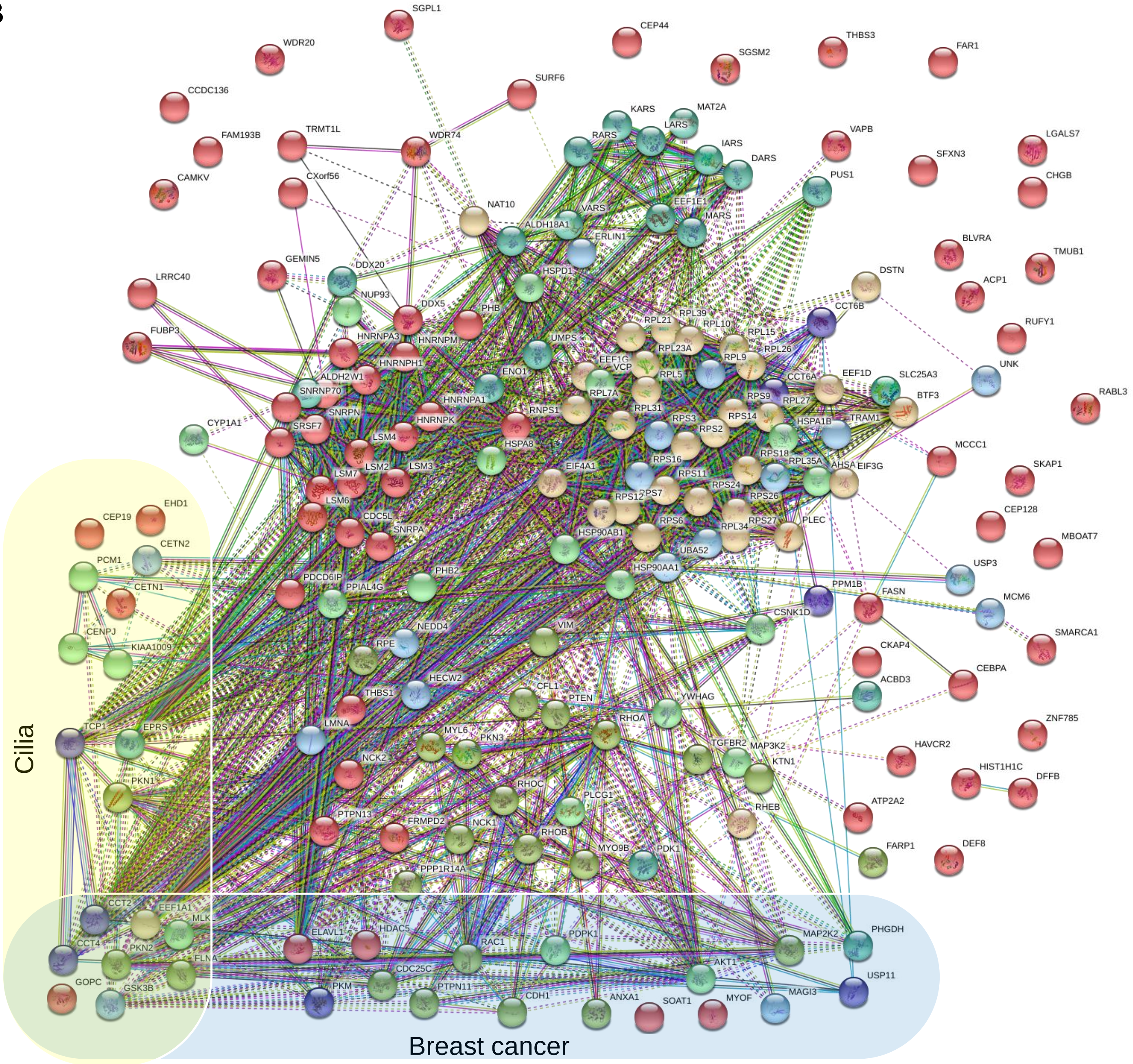

Supplemental Figure 6

Figure 1F

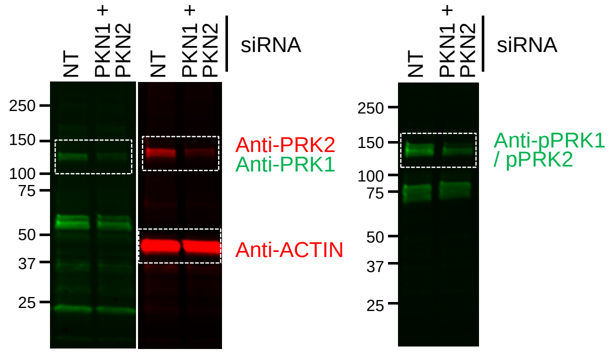

- WB analysis was carried using the LICOR system.
- Images were converted to greyscale using ImageJ.
- The white dashed box indicates the cropped area depicted in the main figure.

Figure 2B

- WB analysis was carried using the LICOR system.
- Images were converted to greyscale using ImageJ.
- The white dashed box indicates the cropped area depicted in the main figure.

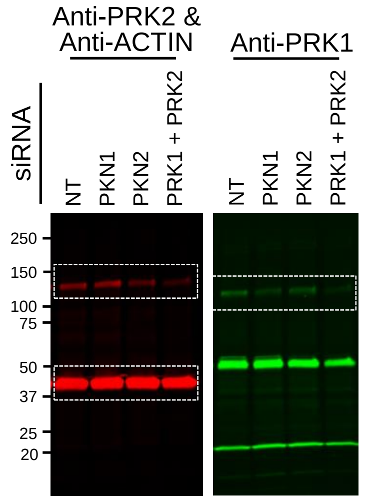

Figure 2A

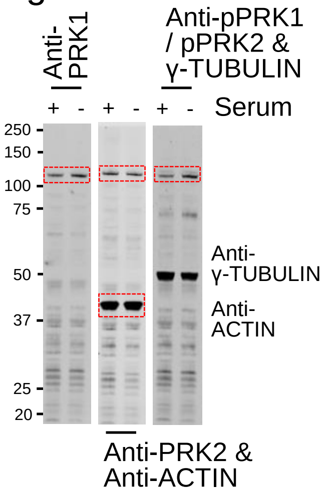

- WB analysis was carried using the LICOR system.
- Images were converted to greyscale using ImageJ.
- The red dashed box indicates the cropped area depicted in the main figure.

Figure 2D

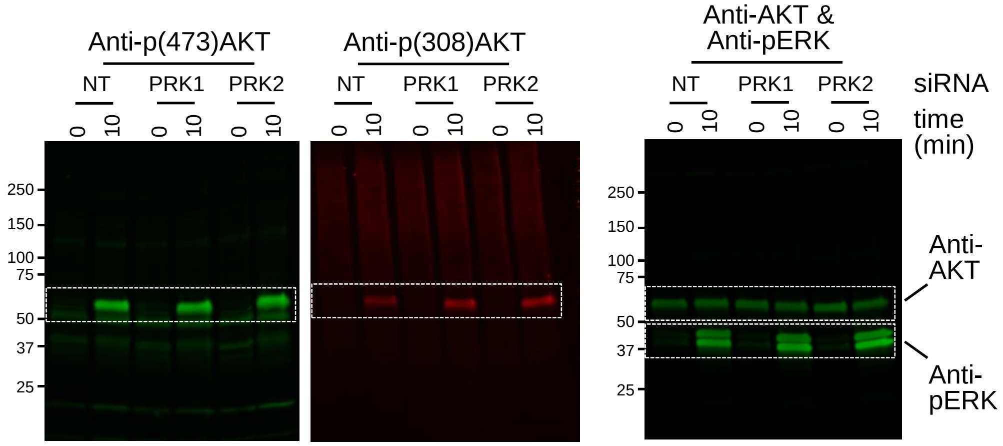

- WB analysis was carried using the LICOR system.
- Images were converted to greyscale using ImageJ.
- The white dashed box indicates the cropped area depicted in the main figure.

Figure 5D

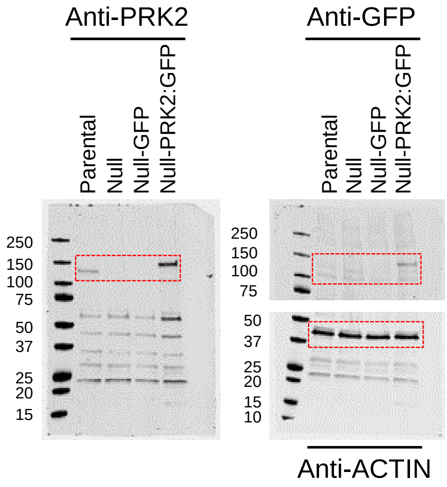

- WB analysis was carried using the LICOR system.
- Images were converted to greyscale using ImageJ.
- The red dashed box indicates the cropped area depicted in the main figure.

Figure 5B

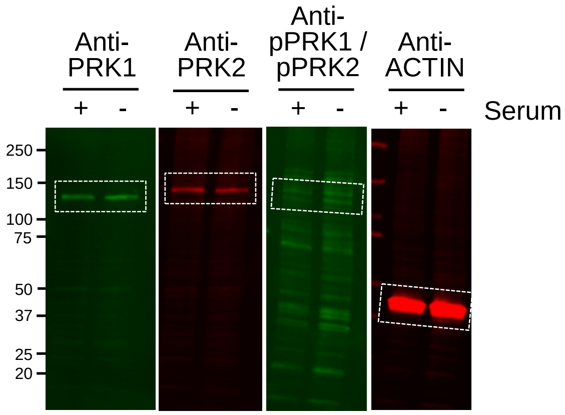

- WB analysis was carried using the LICOR system.
- Images were converted to greyscale using ImageJ.
- The white dashed box indicates the cropped area depicted in the main figure.

Supplementary Figure Legends

Supplemental Figure 1 **A** Sequence similarity between AGC kinases and selected other kinases. **B** Schematic representation of the domains present in the 3 PRK isoforms (top) and the percentage of sequence identity between each other (table). **C** Proteins known to interact with PRK1 and PRK2 (thebiogrid.org). **D** Localisation of pPrk1/pPRK2 at the base (transition zone) of cilia in mIMCD3 cells (scale bar represents 2 µm).

Supplementary Figure 2 **A** Western blot analysis showing a representative co-immuno-precipitation study with PRK1, PRK2 and IgG (control).

Supplementary Figure 3 **A** Western blot analysis showing the extent of PRK1 and PRK2 depletion upon doxycycline induction in the mIMCD3 cells expressing doxycycline inducible shRNA to PRK1 and PRK2, respecitivey. **B** An alternative plot showing the frequency of spheroid sizes in the different shRNA (indicated) inducible mIMCD3 cell lines in the presence and absence of doxycycline.

Supplemental Figure 4 **A** Western blot analysis showing the extent of PRK2 depletion in the indicated TNBC cells. Blots were probed with both PRK2 and Actin (indicated).

Supplemental Figure 5 **A** All proteins known to interact with PRK2. The proteins present in thebiogrid.org (shaded yellow), those identified in this study (shaded blue) and those present in string-db.org (shaded orange) are shown. Black boxed protein (EEF1A1) represents a PRK2 interaction linked to cilia independently identified in this and another study. Grey boxes represent cilia associated PRK2 interacting proteins. **B** All PRK2 interacting proteins were entered into string-db.org (with kmeans clustering set at 7) and known cilia and breast cancer associated proteins separated out to the periphery for visualization purposes.

Supplemental Figure 6 Full length blots of the western blots used in the main Figures.
